# Analysis of Volatile Compounds, Composition, and Thermal Behavior of Coffee Beans According to Variety and Roasting Intensity

**DOI:** 10.3390/foods11193146

**Published:** 2022-10-10

**Authors:** Thomas Dippong, Monica Dan, Melinda Haydee Kovacs, Emoke Dalma Kovacs, Erika Andrea Levei, Oana Cadar

**Affiliations:** 1 Faculty of Science, Technical University of Cluj-Napoca, 76 Victoriei Street, 430122 Baia Mare, Romania; 2 National Institute for Research and Development of Isotopic and Molecular Technologies, 67-103 Donath Street, 400293 Cluj-Napoca, Romania; 3 Research Institute for Analytical Instrumentation, National Institute for Research and Development in Optoelectronics INOE 2000, 67 Donath Street, 400293 Cluj-Napoca, Romania

**Keywords:** coffee, thermal behavior, volatile organic compounds, chemical composition, variety, roasting intensity

## Abstract

This study aimed to investigate the ways in which the thermal behavior, composition, and volatile compound contents of roasted coffee beans depend on variety and roasting intensity. The thermal analysis revealed various transformations in coffee composition, namely, drying, water loss, and decomposition of polysaccharides, lipids, amino acids, and proteins. The results showed that volatile compounds are released differently in coffee depending on coffee type and degree of roasting. The most abundant volatile compounds present in the samples were 2-butanone, furan, 2-methylfuran, methyl formate, 2.3-pentanedione, methylpyrazine, acetic acid, furfural, 5-methyl furfural, and 2-furanmethanol. The total polyphenol contents ranged between 13.3 and 18.9 g gallic acid/kg, being slightly higher in Robusta than in Arabica varieties and in more intensely roasted beans compared to medium-roasted beans. The Robusta variety has higher mineral contents than Arabica, and the contents of most minerals (K, Ca, Mg, Fe, Cu, P, N, and S) increased with roasting intensity. Discrimination between coffee varieties and roasting intensities is possible based on mineral and polyphenol contents.

## 1. Introduction

Coffee is one of the most consumed beverages worldwide and has positive effects on health [1,2]. Coffee consumption is attributed to the psychological stimulation induced by caffeine, the sensory pleasure given by coffee aroma and flavor, and the beneficial health effects [3]. Moderate coffee consumption has been reported to have health benefits, such as reduction in the incidence of several chronic disorders, including diabetes [4], hypertension, and Alzheimer’s and Parkinson’s diseases [5,6,7,8], as well as liver and kidney pathology [9,10,11,12,13]. Moreover, the identification of polyphenols and antioxidants in coffee has further enhanced its consumption. The degree of loyalty to a specific coffee brand depends on external factors, such as label, brand, packaging, price, and marketing, and on consumers’ emotional responses to coffee taste. Therefore, research on the quality parameters that define the sensory quality of coffee, i.e., volatile organic compounds (VOCs), has increased in recent decades [14,15].

According to the International Coffee Organization, worldwide coffee-bean production in 2020 was about 99 million bags of Arabica and 70 million bags of Robusta [16]. The most common and economically important coffee varieties are Arabica and Robusta, representing over 90% of global coffee production [6]. Based on its sensory quality, Arabica (*Coffea arabica* L.) is classified into strictly soft, soft, barely soft, hard, rioysh, rio and rio zona. In contrast, Robusta (*Coffea canephora*) has woody and earthy flavors and is classified into four categories: excellent, good, regular, and abnormal [6,17]. The Arabica varieties are considered to have better sensory characteristics and, consequently, higher purchasing prices than the Robusta varieties [2,6].

Green coffee contains insoluble polysaccharides (~50%), soluble carbohydrates (6–12%), lipids (8–18%), proteins and free amino acids (9–12%), minerals (3–5%), and polyphenolic compounds [1,7,8,18]. The most important alkaloid in green coffee is caffeine, the content of which strongly depends on variety and growing conditions [1,2,6,7,18]. Green-bean composition varies according to coffee variety, agricultural practices, climate, terroir, harvest, and post-harvest practices. The roasting of green beans is the critical step that determines the composition of the roasted beans, although grinding and storage may also influence composition.

The influence of coffee species and varieties, geographical origins, harvesting, roasting, grinding, and brewing on the quality of coffee, from raw beans to the final cup of espresso coffee, has been extensively studied [19,20]. Coffee quality depends on many factors, such as the production system, the chemical composition of the green or roasted beans, the roasting process, and brewing [17,20,21].

The multiple chemical reactions (Maillard reaction, caramelization and Strecker degradation, polyphenol degradation, polymerization of carbohydrates, and pyrolysis) that occur during roasting affect the color, flavor, and aroma of coffee [17,20,22]. During roasting, the formation of undesirable or toxic compounds, such as polycyclic aromatic hydrocarbons and acrylamide, is also possible [22]. Grinding roasted coffee beans is crucial to releasing volatile organic compounds in the brewing of coffee [19]. The main constituents of coffee can also be affected by roasting conditions, ground-particle size, and brewing methods [20].

The composition of coffee allows for differentiation between varieties and the identification of geographical origins and production processes [23]. The content of elements reflects local environmental conditions, such as weather and soil composition, as well as added fertilizers and pesticides [24,25]. Major and trace elements do not decompose during the roasting process; thus, their use as tracers for the authentication of provenance is easier than the use of organic compounds, which may be degraded by roasting temperatures [26]. Due to their water solubility, the minerals will be transferred almost entirely to the final beverage [24].

The volatile organic compounds belonging to different chemical classes identified in roasted coffee, such as alcohols, aldehydes, esters, furans, ketones, phenols, pyrazines, pyridines, pyrroles, and sulfur compounds, define the sensory properties of coffee [19,20]. Some chemicals, such as furans, may harm human health [19]. Volatile organic compounds with low odor thresholds make significant olfactory contributions to coffee flavor [27]. Volatile thiols are among the compounds that impact coffee quality and flavor most, even at very low concentrations [1]. Polyphenols are an essential source of dietary antioxidants and are widely present in fruits, vegetables, cereals, and beverages derived from plants, such as red wine, tea, cocoa, and coffee [18,28]. The roasting temperature of coffee beans noticeably affects the polyphenolic profile through the Maillard reaction, while providing coffee with its pleasant aroma and taste [18]. Additionally, coffee is an essential source of major elements and micronutrients, such as K, Ca, P, Fe, Mn, Zn, and Cu [7].

Data available in the literature may indicate to consumers the beneficial nutritional and health effects, along with the potentially toxic effects, of coffee consumption, as well as information on the available coffee processing methods. Moreover, coffee producers may use data in the literature to explore the relationships between production processes and coffee quality, as well as possible methods for identifying adulteration, while scientists may use the available data to develop new methods for coffee characterization and new ways to fingerprint the authenticity of coffee [29,30].

Many studies have reported on the formation of coffee flavor, mineral and antioxidant contents, and toxic compounds in coffee beans of different varieties subjected to different roasting degrees. However, only a few papers have investigated the volatile organic compound, mineral, and polyphenol contents of coffee beans according to variety and roasting degree. This study presents variations in thermal behavior, volatile organic compound profiles, and mineral and polyphenol contents in commercially available coffee samples of different varieties subjected to different roasting intensities.

## 2. Materials and Methods

### 2.1. Coffee Varieties and Sample Preparation

Six types of roasted coffee beans from recognized coffee brands in original 250–1000 g packages were purchased from specialized markets in Baia-Mare: C1 and C6 (Arabica dark-roast beans (C1, C6); Robusta medium–dark-roast beans (C2); a mix of Arabica and Robusta, medium–dark-roast beans (C3); and Arabica medium-roast beans (C4, C5). We assumed that the information provided on the labels regarding coffee variety and roosting intensity was accurate and that the investigated coffee beans were not adulterated. The coffee beans were dried in an oven at 40 °C and then ground.

### 2.2. Thermal Analysis

The thermal behavior of the ground coffee was investigated by thermogravimetry (TG) and differential thermal analysis (DTA) using a SDTQ600 type instrument (TA Instruments, New Castle, DE, USA) in air and argon atmospheres, up to 1000 °C, at 10 °C/min heating rate, using alumina standards.

### 2.3. Chemical Analysis

For the analysis volatile organic compounds (VOCs) by headspace solid phase microextraction gas chromatography–mass spectrometry (HS-SPME GC-MS), 3 g of ground coffee was transferred to a 20 mL headspace vial (ThermoFischer Scientific, Waltham, MA, USA) and 3 mL of NaCl saturated solution was added to enhance the volatile organic compounds in the headspace and to inhibit any enzymatic reactions. The headspace vials were sealed with crimp-top caps with TFE-silicone headspace septa (ThermoFischer Scientific, Waltham, MA, USA). Each vial was incubated for 20 min at 60 °C. After that time, the solid phase microextraction (SPME) fiber Divinylbenene/Carboxen/Polydimethylsiloxane (50 µm DVB/30 µm CAR/30 µm PDMS) was exposed for 15 min (60 °C) at the headspace of the sample to perform the headspace solid phase microextraction (HS-SPME) extraction of volatile organic compounds. Further, the extracted volatile organic compounds were desorbed for 7 min from the fiber coating into the gas chromatograph (Trace 1310 GC, ThermoFischer Scientific, Waltham, MA, USA) injection port set at 250 °C. The volatile organic compounds were separated using a DB-WAX capillary column (30 m × 0.25 mm i.d. × 0.25 µm film thickness, J & W Scientific Inc., Folsom, CA, USA). Ultrahigh-purity helium was used as a carrier gas at a linear velocity of 1 mL·min^−1^. The oven temperature program was as follows: initial temperature of 35 °C, heated to 180 °C at a rate of 5 °C·min^−1^, increased to 230 °C at a rate of 15 °C·min^−1^, and then held for 7 min. Mass spectra were recorded in electron impact (EI) ionization mode at 70 eV using a mass spectrometer (TSQ 9000 MS, ThermoFischer Scientific, TSQ 9000 MS, ThermoFischer Scientific USA). The quadrupole mass detector, ion source, and transfer-line temperatures were set at 150, 230, and 280 °C, respectively. Mass spectra were scanned in the range of *m*/*z* 50–450 amu. VOCs were identified by comparing the mass spectra with the NIST 14 database system library and linear retention index. The criteria for compound identification required a mass-spectrum matching score of ≥80%. The results were expressed as a percentage of the relative peak area of a peak for each coffee sample, calculated by dividing the peak area by the total peak area of all identified peaks in each chromatogram. The total ion chromatogram (TIC) of each sample was used for peak-area integration.

The minerals (Na, K, Ca, Mg, Fe, Cu, Mn, Zn, and P) were measured using an inductively coupled plasma optical emission spectrometer (Optima 5300DV, ICP-OES, Perkin Elmer, Norwalk, CT, USA) after digestion in a closed-vessel Xpert system (Berghof, Eningen, Germany). Ground coffee-bean samples of 500 mg were digested using 5 mL HNO_3_ 65% and 5 mL H_2_O_2_ 30% in polytetrafluoroethylene digestion vessels, using a four-step digestion program (145 °C and 200 °C—heating; 100 °C and 25 °C—cooling) for a total digestion time of 25 min. Afterward, the vessels were cooled down, and the volume was made up with ultrapure water to 25 mL. The calibration standards were prepared from ICP multi-element standard solution IV, 1000 mg/L (Merck, Darmstadt, Germany), excepting P, for which a mono-element standard solution, 1000 mg/L (Merck, Darmstadt, Germany), was used. All chemicals used were of analytical grade and were purchased from Merck, Darmstadt, Germany. Ultrapure water from a Purelab flex 3 system (Buckinghamshire, UK) was used to dilute the samples and prepare the standard solutions. The total carbon (C), hydrogen (H), nitrogen (N), and sulfur (S) content was carried out using a Flash 2000 CHNS/O analyzer (Thermo Fisher Scientific, Waltham, MA, USA). Polyphenols were measured by the Folin–Ciocalteu colorimetric method using a Lambda 25 spectrophotometer (Perkin-Elmer, Waltham, MA, USA) to measure the blue complex at 760 nm with gallic acid as a reference standard [31]. All measurements were performed in triplicate, and data were expressed as the means ± standard deviations.

### 2.4. Data Analysis

The differences in the elemental contents of different coffee varieties were tested by comparing the averages of the three replicates using the Tukey test for a significance level of 0.05, using OriginPro (version 2020b) software (OriginLab Corporation, Northampton, MA, USA). The coffee-bean samples were grouped according to elemental compositions and polyphenol contents by Agglomerative Hierarchical Clustering (AHC) using the squared Euclidian distance and the Ward method for combining clusters, using XLStat software version 2019.3.2 (Addinsoft, Paris, France).

## 3. Results

### 3.1. Thermal Behavior

**Figure 1:**
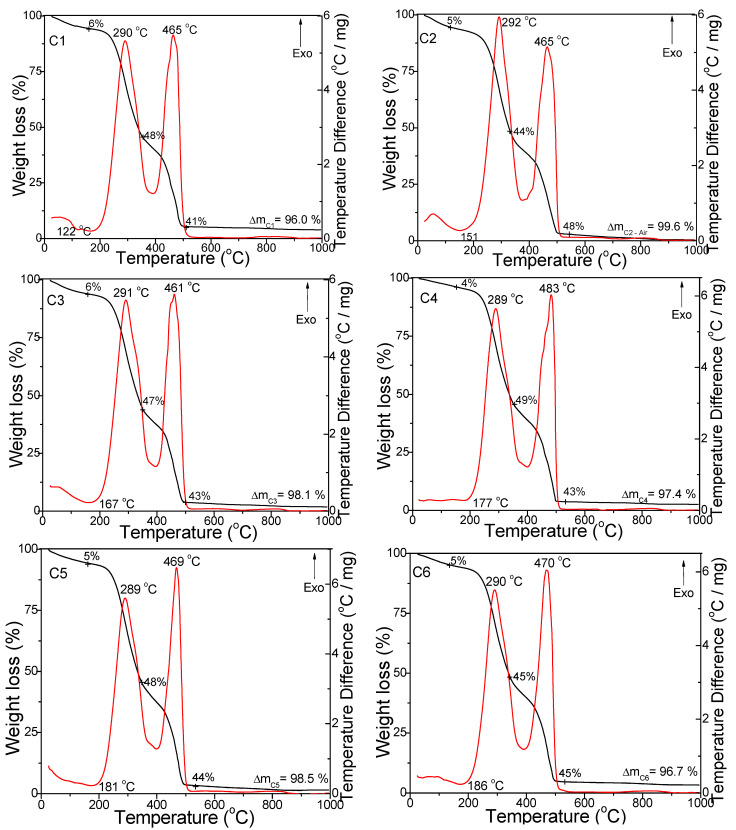
TG (black line) and DTA (red line) curves of the coffee samples under air atmosphere.

**Figure 2:**
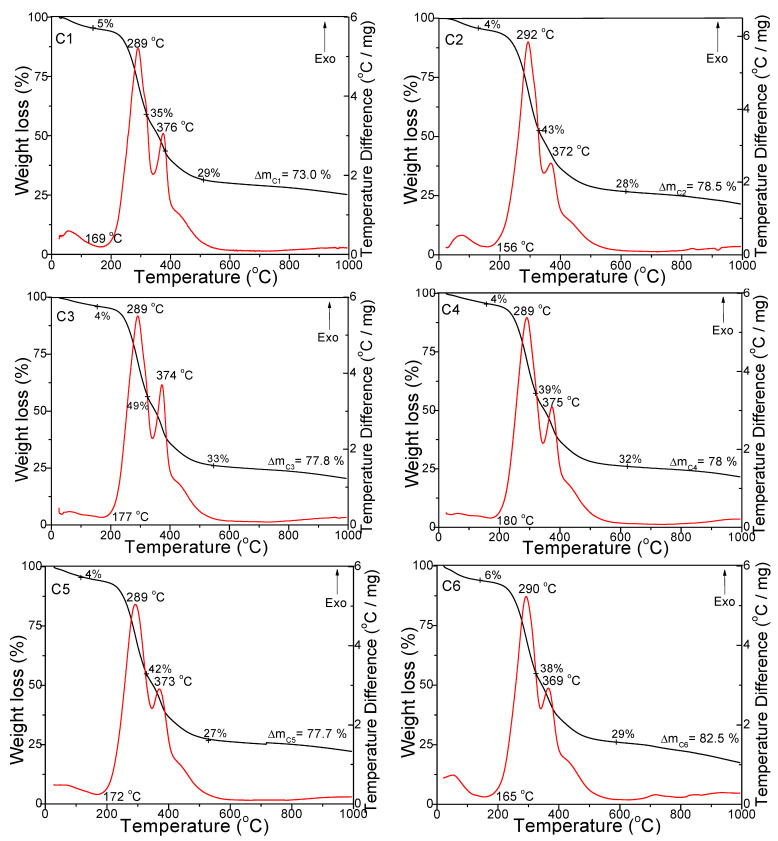
TG (black line) and DTA (red line) curves of the coffee samples under argon atmosphere.

The decomposition stages of the coffee samples were investigated in air (Figure 1) and argon atmospheres (Figure 2) up to 1000 °C. In all cases, the DTA curves showed an endothermic effect between 122 and 186 °C associated with a 4–6% mass loss on the TG curve, an exothermic effect in the range of 289–292 °C associated with a mass loss of 35–49% on the TG curve, and a second wide exothermic effect on the DTA curve at 461–483 °C (air) and 369–376 °C (argon) associated with a mass loss of 41–48% (air) and 27–33% (argon) on the TG diagram. The peaks under argon atmosphere had lower intensities and appeared at lower temperatures than under air atmosphere. The total mass loss was also lower under argon atmosphere (73.0–82.5%) compared to air atmosphere (96.0–99.6%).

### 3.2. HS-SPME GC-MS Analysis of Volatile Organic Compounds

**Figure 3:**
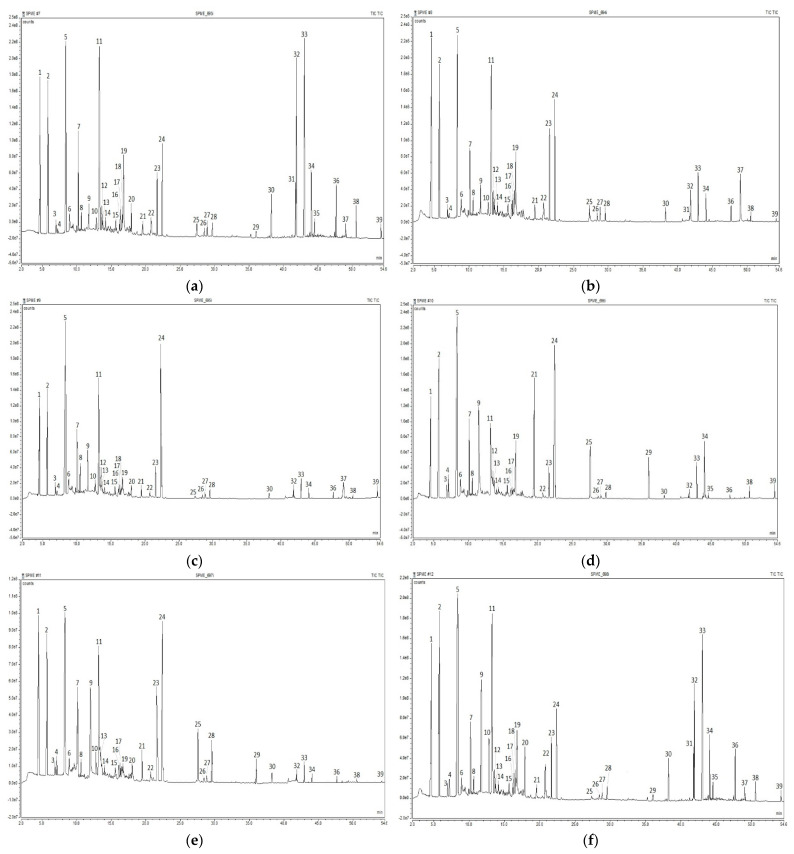
Total ion chromatograms of volatile organic compounds identified in coffee samples by HS-SPME-GC-MS analysis: (**a**) C1; (**b**) C2; (**c**) C3; (**d**) C4; (**e**) C5; and (**f**) C6.

A total of 39 volatile organic compounds were identified in the studied coffee samples (Table 1) by HS-SPME GC-MS analysis. The mass-spectra matching score was ≥80% for most of the compounds, except for 2-oxopropanal, 2-butanol, 3-pentanone, 2-acetylfuran, and 2-acetyl-5-methylfuran, for which the matching score was ≥74%. Twelve compounds belonged to furans, eight to ketones, five to aldehydes, four to alcohol and pyrazines, two to pyrroles, and one to esters, oxazoles, carboxylic acid, and phenolic compounds, respectively. Total ion chromatograms of the analyzed samples are provided in Figure 3. The odor characteristics were compared with those presented in [32].

**Figure 4:**
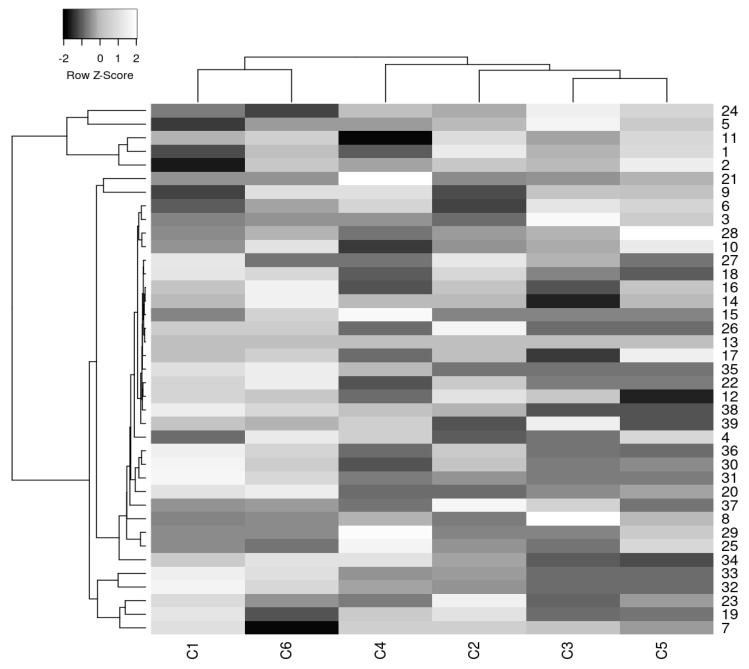
Hierarchical cluster analysis through heatmapping of coffee samples.

Hierarchical cluster analysis through heatmapping (Figure 4) was conducted to find the inter-connectivity and closeness of the studied coffee samples and individual volatile organic compounds. Heatmap representation clearly showed differentiation with respect to the volatile aromatic-compound profiles of the samples. At the same time, the hierarchical clustering indicated that C1 and C6 samples (dark-roasted Arabica varieties) were distributed separately and fell aside from the heatmap compared with the other samples (medium-roasted Arabica, Robusta, and their mixtures).

### 3.3. Mineral Compositions and Total Polyphenol Contents

The contents of several macro- and microelements and polyphenols along with the elemental compositions of the coffee beans are presented in Table 2.

## 4. Discussion

### 4.1. Thermal Behavior

The endothermic effect between 122 and 186 °C was attributed to the drying of coffee powders and the desorption of physically absorbed water molecules (dehydration). The broad exothermic effect that appeared in the range of 289–292 °C on the DTA curves was ascribed to polysaccharide and lipid decomposition, while the second wide exothermic effect on the DTA curve, present for all samples at 461–483 °C (air) and 369–376 °C (argon), was attributed to the decomposition of amino acids and proteins [27].

### 4.2. Volatile Organic Compounds

The compositions of volatile organic compounds in green and roasted beans differed considerably. The most important aromatic compounds are formed during roasting. Green beans do not contain coffee aromatic compounds, though they have similar groups of compounds.

Furans were the most abundant group of volatiles present in the coffee samples, their contents being higher than 40%, although their amounts varied between samples, as follows: C3 (57.1%) > C5 (50.8%) > C1 = C6 (49.4%) > C2 (48.3%) > C4 (44.5%). The same volatile organic compounds were reported in studies on roasted coffee beans [21,33,34,35] and brewed coffee [36].

Furans are cyclic ethers mainly found in condensates from carbohydrates that undergo browning reactions. As green coffee beans contain large amounts of sugars, mainly sucrose, these compounds are formed through thermal processes, such as coffee roasting, when degradation and rearrangement of carbohydrates, ascorbic acid, and unsaturated fatty acids occur [19,37,38]. The thermal degradation of d-glucose and sugar polymers into furanic compounds as major decomposition products was also reported by Kim et al. [39]. Serine, threonine, and sucrose decompose into heterocycles as simple furans, furanones, and furan rings. Our data (Table 1) align with reports in the literature that roasted Arabica coffee beans have higher contents of stream-volatile furans than the Robusta variety because of the high sucrose contents [40,41]. Although the presence of furans in thermally treated foods, such as roasted coffee, is mainly attributed to carbohydrates resulted in Maillard-reaction, it could also result from the thermal oxidation of lipids, thiamine degradation, and higher nucleotide or terpene breakdown [42]. The 2-methylfuran was the most prevalent furan compound detected in the samples (13.7–22.8%), except for the C1 sample, in which 2-furanmethanol was the dominant (11.6%) volatile compound. This compound was previously identified in both green and roasted coffee [39,43]. Furfural (19.6–4.7%) and furans (13.7–5.8%) were also found in high amounts in all samples, being responsible for the chocolatey, caramelly, nutty, creamy, bready, and ethereal odors of coffee.

The second prevalent group of compounds identified in coffee samples was ketones, which also constitute an essential class of volatile compounds. They represent about 20–27% of coffee aromatic components, having as representatives 2,3-pentanedione and 2-butanone. As in the case of furans, unroasted coffee beans do not contain these compounds. They are formed through roasting when the Maillard reaction leads to diketone formation following the heating of the glucose in the coffee beans. They also were identified as the representative aliphatic volatiles formed after the thermal interaction of glucose and cysteine [43]. According to studies in the literature, ketone amounts increase with roasting temperature and duration [44]. Similar to the other compounds, even ketone amounts differed among the studied samples.

Pyrazines are also characteristic aromatic compounds specific to roasted coffee. Methylpyrazine was identified in all the studied coffee samples, the highest amounts being detected in C1, C2, and C4 (>4% of the total detected volatile organic compounds). Pyrazines provide various odors, such as nutty, roasted, corn, hazelnut, potato or earthy aromas, depending on the substituents of the pyrazine ring; pyridine is related to bitter, fishy or burnt odors, while pyrrole provides nutty notes [19].

Pyrroles are primarily formed thermally; they are not present in fresh or raw foods, excepting 1-methylpyrrole and 2-acetylpyrrole, which have been identified in green coffee [41]. Pyrroles are formed by the reaction of aldoses with alkylamines, in a reaction involving reducing sugars and amino acids by condensation of glucose and alanine, or of glucose and proline or hydroxyproline [41]. These compounds have nutty, hay-like, and herb aromas [45]. In our samples, the identified pyrroles (1-ethyl-2-pyrrolecarbaldehyde and 2-formyl-1-methylpyrrole) were responsible for the roasted aromas. Their amounts ranged within 0.2–4%, with clear differentiation between the studied samples. The average values of aldehydes and alcohols were 4 and 5%, respectively.

Low contents of aldehydes were identified in the C2 samples. Aldehydes are formed either through the oxidative degradation of amino acids during their interaction with sugars at high temperatures or through the interaction of amino acids and polyphenols in the presence of polyphenol oxidase at normal temperatures [46]. Benzaldehyde was identified in the highest amounts in C1, C2, C4, and C6 samples (≥1.5%) and is often associated with the oxidation of unsaturated fatty acids through the breakdown of hydroperoxide intermediates. Their presence was not surprising, as coffee beans are known to contain lipids and proteins. In the literature, the total lipid content is around 13% in Arabica coffee and around 10% in Robusta, and of these contents, nearly half are represented by linoleic acid (C18:2). In the volatile flavor compounds formed by the autoxidation of linoleic acid and methyl linoleate, hexanal was identified most frequently. According to our experimental data, hexanal represented less than 1%, in accordance with the results reported by Toci et al. [34] and Budryn et al. [47]. Benzaldehyde and 2,3,5,6-tetramethylpyrazine may be considered markers of defective beans [36]. Generally, aldehydes participate in the staling of roasted coffee in the presence of atmospheric oxygen. Stale notes in roasted coffee were correlated with the generation of hexanal after long-term storage [36].

Guaiacol, a common phenolic compound in coffee samples, is produced by carbohydrate catabolism and is connected with the spicy and smoky notes of aromatic coffee [19,21]. This aromatic compound was detected in the C1, C4, and C6 samples.

The volatile acids in coffee are responsible for the flavor constituents, while the less volatile or non-volatile ones contribute to the taste quality [43]. Acetic acid was the only carboxylic acid identified with high contents, especially in the C1 (5.8%) and C2 (7.3%) coffee samples. Roasting temperature, as well as roasting duration, control the development of the aroma [22].

The roasting conditions, as well as coffee variety, climate, and terroir, highly influence the volatile compounds in coffee [20]. Generally, the differences between Arabica and Robusta coffee are evident due to the different compositions of aromatic precursors and the comminution processes determined by the different hardness of beans [19]. The differences in roasting profiles and the more aggressive roasting conditions employed for Robusta than for Arabica result in reduction of volatile organic compound intensity in Robusta [19].

### 4.3. Mineral Compositions and Total Polyphenol Contents

The macroelement content decreased in the order K >Mg > P > Ca > Na, while the microelement content decreased in the order Fe > Mn > Cu > Zn. A similar trend in macro- and microelement contents was reported in green coffee beans from different regions of Ethiopia [48]. The highest macro- and microelement, N, C, H, and polyphenol contents, were found in Robusta medium–dark-roast beans (C2), while the lowest were found in Arabica medium-roast beans (C4), excepting Na and Zn. The differences between the highest and the lowest contents of each macro- and microelement were between 22 and 50%, around 50% for N and S, 30% for polyphenols, and 3% for C, indicating that coffee production influences the composition of the final product. Based on their mineral contents and the almost complete transfer of minerals from coffee beans to final beverages, the studied coffee varieties are possible sources of minerals in the human diet [2,8,24,48].

At the same roasting intensity, the Robusta variety has higher contents of Na, K, Ca, Mg, Fe, Cu, Mn, Zn, P, N, C, S, and polyphenols than the Arabica variety. In the case of Arabica, with increasing roasting intensity, K, Ca, Mg, Fe, Cu, P, N, S, and polyphenol contents increase, while contents of Na, Mn, Zn, C, and H are not influenced by roasting intensity. The differences in the elemental concentrations might be due to factors related to variety, soil type, fertilizers, and pesticides used in cultivation, as well as technological processes [7]. Similar contents of macro- and microelements in ground coffee beans were reported by Janda et al. [8] for coffee brews made and served in a coffee shop in Poland. The high variability of major elemental contents allowed for geographical differentiation between roasted Arabica coffee samples produced in different regions of Mexico with good accuracy [23].

Polyphenols are linked to health-promoting properties, such as antioxidant, anti-inflammatory, antidiabetic, and anticarcinogenic activities [8]. The content of antioxidant compounds is also influenced by the roasting process [46]. Polyphenols are thermolabile molecular structures that decompose during drying and roasting due to the high temperatures and enzymatic browning processes. The highest content of polyphenols was measured in Robusta medium–dark-roast beans (C2, 18.9 g GA/kg), the lowest in Arabica medium-roast (C4, 13.3 g gallic acid/kg), which indicates that the content of polyphenols slightly increases with the roasting process and is higher in Robusta than in Arabica species. Similarly, Vignoli et al. reported higher phenol contents in roasted Robusta beans than in roasted Arabica beans [49]. Oppositely, decrease in total polyphenols with increase in roasting intensity and higher total polyphenol contents in Arabica as compared with Robusta species were reported by Hasbullah and Umiyati [46]. The opposite results of different studies may be attributed to the different origins of the studied coffee beans, their different climates and soils leading to different levels of polyphenol contents in the green beans [46,50].

Generally, the Robusta variety (C2) had slightly higher elemental contents, although, except for Cu and polyphenols, the differences were not significant at a probability of 0.05 compared to the other coffee samples. The dark-roast Arabica coffee beans (C1, C6) had significantly higher K, Ca, N, and S contents than the medium-roast Arabica beans (C4, C5). However, the other elements varied without a clear link with roasting intensity, depending on other conditions, such as origin, soil characteristics, etc. Except for the polyphenol contents being slightly higher in the medium–dark-roast Robusta than in the medium–dark-roast mixture of Robusta and Arabica, the other elements did not differ significantly between the two samples.

**Figure 5:**
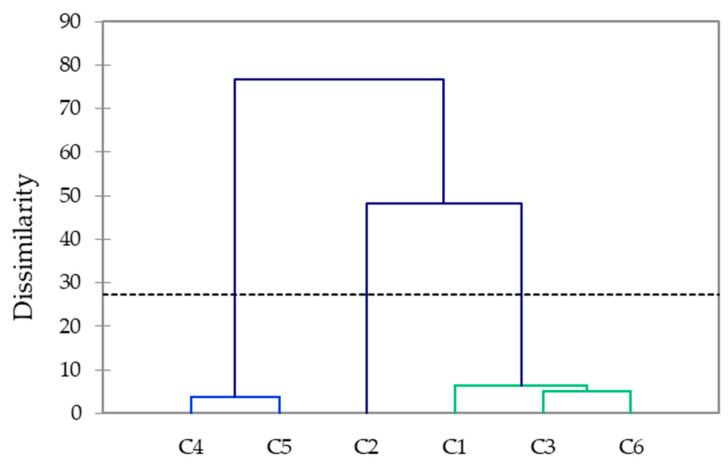
Agglomerative hierarchical clustering of the studied coffee beans based on their macro-, microelement, N, C, H, and polyphenol contents. The dotted line represents automatic truncation, resulting in three groups: group 1 (light blue), group 2 (blue), and group 3 (green).

Based on the elemental compositions and polyphenol contents, AHC (Figure 5) grouped the coffee beans into three clusters. The first cluster included the moderate-roast Arabica beans (C4, C5), the second cluster contained the intense-roast Robusta beans (C1), and the third cluster encompassed the intense-roast Arabica and Arabica–Robusta mixtures (C1, C3, C6).

## 5. Conclusions

Roasting is one of the main processes that causes changes in the chemical properties of coffee. The compositions of volatile organic compounds, minerals and polyphenol contents allowed the discrimination between Robusta and Arabica coffee varieties and different roasting intensities. The studied coffee beans’ thermal behaviors were similar. The DTA curves showed one endothermic and two exothermic effects, attributed to the coffee drying and polysaccharide, lipid, amino acid, and protein decomposition. During the decomposition processes, high mass loss took place. The furans were the dominant volatiles in the samples, followed by ketones, pyrazines, and pyrroles. The proportions of volatiles varied from sample to sample. The minerals and polyphenol contents were lower in the Arabica than in the Robusta variety, and most of the mineral (K, Ca, Mg, Fe, Cu, P, N, and S) contents increased with roasting intensity. However, the roasting intensity did not influence Na, Mn, Zn, C, or H contents. Due to the high mineral contents and the presence of polyphenols, coffee is a good mineral source for the human diet.

## Figures and Tables

**Table 1:** Volatile organic compound contents (%) identified by HS-SPME GC-MS.

Peak No.	Compound Name	Group	Odor Characteristic	Samples					
				C1	C2	C3	C4	C5	C6
1	2-Butanone	Ketones	Ethereal	6.2	12.5	8.9	6.7	11.2	9.3
2	Furan	Furans	Ethereal	5.8	10.9	10.1	9.3	13.7	10.9
3	2-Oxopropanal	Aldehydes	Caramelly	0.4	0.3	1.5	0.5	0.9	0.5
4	2-Butanol	Alcohol	Fruity	0.2	0.1	0.3	1.3	1.5	1.9
5	2-Methylfuran	Furans	Chocolatey	9.3	15.2	22.8	13.8	16.8	13.7
6	2-Methylbutanal	Aldehydes	Chocolatey	0.5	0.4	1.4	1.2	1.2	0.8
7	Methyl formate	Esters	Fruity	6.7	5.8	5.2	5.8	4.2	1.3
8	2,3-Butanedione	Ketones	Buttery	0.5	0.4	3.6	1.1	1.2	0.6
9	2-Methyl-3-buten-2-ol	Alcohol	Herby	0.7	0.9	4.2	6.4	4.1	6.4
10	3-Pentanone	Ketones	Ethereal	0.4	0.4	0.5	0	1.2	1.1
11	2,3-Pentanedione	Ketones	Buttery	9.1	11.2	8.8	5.7	10.8	10.3
12	Hexanal	Aldehydes	Green	0.6	0.7	0.5	0.2	0	0.5
13	2-Methyl-2-butenal	Aldehydes	Green	0.2	0.2	0.2	0.2	0.2	0.2
14	3-Penten-2-one	Ketones	Fruity	0.3	0.3	0.2	0.3	0.3	0.4
15	3,4-Hexanedione	Ketones	Buttery	0.3	0.3	0.3	0.5	0.3	0.4
16	2-Methyl-1-butanol	Alcohol	Roasted	0.3	0.3	0.2	0.2	0.3	0.4
17	Furfuryl methyl ether	Furans	Coffee	0.4	0.4	0.1	0.2	0.7	0.5
18	Trimethyloxazole	Oxazoles	Nutty	0.5	0.4	0.1	0	0	0.4
19	Methylpyrazine	Pyrazines	Nutty	6.3	6.1	1.3	4.2	1.5	0.7
20	3-Hydroxybutanone	Ketones	Buttery	2.6	0	0.4	0	0.8	3.1
21	1-Hydroxy-2-propanone	Ketones	Caramelly	0.4	0.3	0.5	9.7	1.6	0.5
22	3-Methyl-2-buten-1-ol	Alcohol	Fruity	0.6	0.5	0.2	0.1	0.2	0.8
23	Acetic acid	Carboxylic acid	Acidic	5.8	7.3	2.4	2.9	3.5	3.3
24	Furfural	Furans	Bready	7.7	10.3	19.6	11.8	15.5	4.7
25	1-Ethyl-2-pyrrolecarbaldehyde	Pyrroles	Roasted	0.4	0.5	0.1	3.2	2.1	0.1
26	2-Acetylfuran	Furans	Balsamic	0.2	0.3	0.1	0.1	0.1	0.2
27	Furfuryl ether	Furans	Coffee	0.5	0.5	0.3	0.2	0.2	0.2
28	5-Ethyl-2.3-dimethylpyrazine	Pyrazines	Burnt	0.4	0.5	0.6	0.3	1.6	0.6
29	Furfuryl propionate	Furans	Fruity	0.2	0	0	3.3	1.3	0.2
30	Furfuryl acetate	Furans	Fruity	2.3	1.1	0.4	0.1	0.5	1.3
31	2-Ethyl-2.3-dimethylpyrazine	Pyrazines	Nutty	2.7	0.2	0	0	0	1.6
32	5-Methyl furfural	Furans	Caramelly	8.4	1.9	0.7	2.4	0.6	5.8
33	2-Furanmethanol	Furans	Bready	11.6	3.4	1.2	3.1	1.2	9.6
34	Benzaldehyde	Aldehydes	Fruity	2.6	1.6	0.5	3.7	0.3	3.7
35	Guaiacol	Phenolic compounds	Phenolic	0.5	0	0	0.2	0	0.6
36	γ-Butyrolactone	Furans	Creamy	2.6	1.5	0.3	0.2	0.2	1.8
37	2-Acetyl-5-methylfuran	Furans	Nutty	0.4	2.8	1.5	0	0	0.5
38	2-Formyl-1-methylpyrrole	Pyrroles	Roasted	0.9	0.4	0.1	0.5	0.1	0.7
39	5-Methyl--cyclopentapyrazine	Pyrazines	Earthy	0.5	0.1	0.9	0.6	0.1	0.4

**Table 2:** Mineral, C, H, N, S, and polyphenol contents in the studied coffee beans expressed as averages ± standard deviations (n = 3). Different superscript letters represent significant differences (*p* < 0.05) in each parameter between the coffee-bean samples.

Sample	C1	C2	C3	C4	C5	C6
Na (mg/kg)	68.9 ± 5.5 ^b^	89.4 ± 7.4 ^a^	74.3 ± 6.9 ^ab^	70.1 ± 6.0 ^b^	69.6 ± 6.3 ^b^	64.3 ± 5.6 ^b^
K (mg/kg)	20,034 ± 567 ^ab^	21,536 ± 866 ^a^	20,123 ± 434 ^ab^	16,562 ± 788 ^c^	18,002 ± 880 ^bc^	20,088 ± 984 ^ab^
Ca (mg/kg)	1332 ± 86 ^a^	1411 ± 75 ^a^	1342 ± 84 ^a^	889 ± 60 ^b^	934 ± 63 ^b^	1348 ± 77 ^a^
Mg (mg/kg)	2112 ± 127 ^bc^	2645 ± 117 ^a^	2301 ± 160 ^ab^	1734 ± 114 ^cd^	1815 ± 137 ^d^	2335 ± 150 ^ab^
Fe (mg/kg)	54.3 ± 5.1 ^bc^	69.2 ± 5.6 ^a^	60.7 ± 5.3 ^ab^	35.5 ± 3.6 ^d^	39.6 ± 3.7 ^cd^	58.7 ± 4.8 ^ab^
Cu (mg/kg)	12.3 ± 1.2 ^bc^	24.1 ± 2.3 ^a^	14.9 ± 1.3 ^bc^	11.2 ± 1.1 ^bc^	12.3 ± 1.2 ^c^	16.2 ± 1.5 ^b^
Mn (mg/kg)	20.4 ± 2.2 ^a^	25.3 ± 2.8 ^a^	23.4 ± 2.5 ^a^	19.6 ± 2.1 ^a^	20.1 ± 1.9 ^a^	20.9 ± 1.9 ^a^
Zn (mg/kg)	6.94 ± 0.74 ^b^	8.96 ± 0.92 ^a^	7.08 ± 0.73 ^ab^	7.34 ± 0.69 ^ab^	7.03 ± 0.65 ^b^	5.95 ± 0.59 ^ab^
P (mg/kg)	1524 ± 111 ^b^	1953 ± 150 ^a^	1652 ± 127 ^ab^	1356 ± 101 ^b^	1455 ± 109 ^b^	1863 ± 124 ^a^
C (%)	48.9 ± 0.65 ^a^	50.4 ± 0.68 ^a^	49.6 ± 0.72 ^a^	48.9 ± 0.59 ^a^	49.0 ± 0.67 ^a^	49.4 ± 0.64 ^a^
H (%)	5.78 ± 0.18 ^bc^	6.45 ± 0.20 ^a^	5.94 ± 0.16 ^cd^	5.11 ± 0.16 ^d^	5.68 ± 0.19 ^bc^	5.93 ± 0.17 ^b^
N (%)	2.96 ± 0.10 ^a^	3.05 ± 0.10 ^a^	2.99 ± 0.08 ^a^	1.64 ± 0.06 ^b^	1.76 ± 0.05 ^b^	3.05 ± 0.09 ^a^
S (%)	0.084 ± 0.005 ^b^	0.121 ± 0.007 ^b^	0.094 ± 0.005 ^b^	0.054 ± 0.003 ^c^	0.064 ± 0.003 ^c^	0.088 ± 0.005 ^b^
Polyphenol content (g GA/kg)	14.5 ± 0.5 ^cd^	18.9 ± 0.6 ^a^	16.4 ± 0.7 ^b^	13.3 ± 0.5 ^d^	14.8 ± 0.6 ^cd^	15.7 ± 0.5 ^bc^

## Data Availability

The data presented in this study are available on request from the corresponding author.
